# Cardiovascular Risk Assessment and Its Determinants Among Older Adults in India: Evidence From a Nationally Representative Survey

**DOI:** 10.1002/cdt3.70052

**Published:** 2026-05-03

**Authors:** Vansh Maheshwari, Shyamashree Das, Saurav Basu

**Affiliations:** ^1^ Population Health Sciences Institute Newcastle University Newcastle upon Tyne UK; ^2^ Department of Community Medicine ESI‐PGIMSR, Medical College & Hospital And ODC(EZ) Joka Kolkata India

**Keywords:** cardiovascular risk, India, older adults, WHO

## Abstract

**Background:**

Cardiovascular disease (CVD) is the leading cause of mortality, posing a major challenge to its rapidly aging demographic. Early identification of individuals at high risk for future cardiovascular events is paramount for implementing timely preventative strategies. However, comprehensive population‐level estimates using validated assessment tools remain limited in this setting. This study was to ascertain the prevalence and determinants of persons at high risk of experiencing any cardiovascular event within the next 10 years among older adults in India.

**Methods:**

We analyzed data from the Longitudinal Aging Study in India Wave‐1 (2017–2018), including 64,266 participants aged 40–74 years. We used the World Health Organization CVD Risk Chart 2019 version, a nonlaboratory‐based risk assessment model, to stratify cardiovascular risk based on age, sex, smoking status, systolic blood pressure, and body mass index.

**Results:**

Weighted prevalence of high CVD risk ( ≥ 20%) was 2.67% (95% confidence interval [CI]: 2.44, 2.91), while intermediate risk (10% to < 20%) was 28.41% (95% CI: 27.60, 29.25). Overall, 31.08% had ≥ 10% risk. In the diabetes and hypertension (HTN) subgroups, high risk ( ≥ 20%) was 3.53% and 4.36%, respectively. Adjusted analysis showed significantly higher odds of high risk among alcohol users, individuals with prior HTN, and widowed/separated participants. Conversely, college education and current employment were associated with significantly lower odds.

**Conclusions:**

Over 3 in 100 Indian adults aged ≥ 40 years with diabetes or HTN face a ≥ 20% 10‐year CVD risk, highlighting an urgent need for targeted primary care interventions. Prioritizing alcohol cessation, aggressive blood pressure management, and glycemic control through universal standard care coverage is essential to mitigate this burden.

## Summary

- Nearly one‐third (31.08%) of the surveyed adults aged ≥ 40 face a 10‐year cardiovascular risk of ≥ 10%.
- Over 3% of Indian adults aged ≥ 40 with diabetes or hypertension face a ≥ 20% 10‐year cardiovascular risk.
- Noticeable treatment gaps exist with only 91.81% of diabetic and 79.18% of hypertensive individuals in the highest cardiovascular risk category ( ≥ 30%) currently taking medication.
- Prioritizing alcohol cessation, aggressive blood pressure management, and glycemic control through universal standard care coverage is essential to mitigate this burden.

## 1 Introduction

Noncommunicable chronic diseases (NCDs), including cardiovascular diseases (CVDs), stroke, cancer, chronic respiratory diseases, and diabetes mellitus (DM), account for 74% of all global deaths annually, with 374 million disability‐adjusted life years (DALY). Low‐and‐middle‐income countries (LMICs) experience 77% of NCD‐related deaths [1].

India has been experiencing an epidemiological transition in the 21st century, wherein the burden of NCD is surpassing the burden of communicable diseases. In 2021, India reported 63% of total deaths due to NCD, of which 24.5% (2.8 million) were attributed to CVDs, along with 74 million DALY, with 22% being premature deaths [2]. CVD is also the second leading cause of out‐of‐pocket expenditure in the country with nearly half of the households having people with CVD are at risk of catastrophic health expenditure and resultant poverty [3]. Furthermore, 92.28% of DALYs associated with CVD are attributable to modifiable risk factors, primarily tobacco and alcohol use, physical inactivity, unhealthy diet, obesity, high blood pressure, lipids, and glucose, which can be prevented by identifying high‐risk individuals and implementing appropriate behavioral modifications and health promotion through healthy diet, exercise, tobacco and alcohol cessation [4, 5].

NCD‐related premature mortality reduction target by at least one third till 2030 is a key sustainable development goal (SDG) target whose attainment requires provision of counseling, diagnostics, and drugs to at least half of the population aged ≥ 40 years and those with high CVD risk, which would prevent or delay the incidence of CVD and their associated complications [6]. The World Health Organization's (WHO's) preferred cost‐effective best buy strategy to control CVD, considering the multifactorial etiology of these diseases, is population‐based total risk assessment for stratifying individuals as per their extent of risk of experiencing a CVD event within the next 10 years [4].

Several tools have been developed to assess and manage CVD risk at the population level. Risk prediction charts, such as the Framingham Risk Score, Globorisk, SCORE–European High‐Risk Chart, and the WHO–International Society of Hypertension (ISH) classification, help identify individuals at increased risk of developing CVDs [7]. The updated WHO CVD risk prediction charts from 2019 included a risk‐predictor model with a nonlaboratory version tailored for use in resource‐constrained settings with inadequate laboratories for routine blood glucose and total cholesterol measurement [8]. It has also been previously validated in Indian community‐based settings [9].

Persons with a 10‐year CVD event risk ≥ 20% represent those at high risk having a more than 2 in 10 chance of occurrence of a CVD event within the next 10 years. Identifying individuals at high risk of CVD, especially among individuals with chronic diseases frequently interacting with the health system, provides opportunities for health system strengthening through enhanced screening, identification, and effective therapy to reduce CVD‐related morbidity, mortality, and associated economic losses. Furthermore, DM and hypertension (HTN) are highly prevalent comorbid conditions that frequently co‐occur, synergistically accelerating atherosclerotic pathways [10]. People with DM and HTN, therefore, have higher likelihood of CVD events with further risk accentuation in LMIC settings with high burden of medication nonadherence [11]. Since the WHO nonlaboratory risk score excludes DM, independent evaluation of this comorbidity is essential to avoid underestimating the true cardiovascular vulnerability of diabetic patients.

The present study was therefore conducted to ascertain the prevalence and determinants of persons at high risk of experiencing any cardiovascular event within the next 10 years among older adults in India, with subgroup analysis in patients with DM or HTN, including their treatment status using a nationally representative dataset.

## 2 Methods

### 2.1 Study Design and Participants

This research is based on a comprehensive cross‐sectional secondary analysis of the Longitudinal Aging Study in India (LASI) Wave‐1 data, which was conducted between 2017 and 2018. The LASI Wave‐1 sample size was 73,000 individuals aged ≥ 45 years, along with their spouses (regardless of their age), across various states and union territories of India. A multistage stratified area probability sampling design was used, utilizing the 2011 Census of India as the sampling frame. Primary sampling units were selected, comprising villages in rural areas and wards in urban areas, based on probability proportional to size [12].

### 2.2 Data Collection and Measurements

Data collection for this study included household interviews, individual questionnaires, and physical measurements. The household interviews provided insights into the living conditions, family structure, and economic status of the participants. Individual questionnaires were instrumental in gathering detailed information about lifestyle habits, health status, and personal history, particularly focusing on smoking habits, which is a critical determinant in CVD risk assessment. Physical measurements were conducted by trained medical professionals following standardized protocols. These measurements included blood pressure readings, and body weight and height measurements, necessary for calculating body mass index (BMI). The consistency and accuracy of these measurements were periodically validated to ensure the reliability of the data.

### 2.3 Study Sample

The sample in the present analysis included all individuals aged 40–74 years, regardless of whether they had a prior diagnosis of HTN or DM or whether they were currently taking medication for these conditions. Participants aged < 40 years were purposefully included in this analysis to align exactly with the minimum age threshold established by the WHO CVD Risk Chart. Wave‐1 of LASI included 73,396 participants, from which 9130 individuals aged < 40 or > 74 years were excluded from the analysis. The analysis was thereby conducted on 64,266 participants.

### 2.4 WHO CVD Risk Chart

The 2019 WHO CVD Risk Chart is a nonlaboratory‐based risk assessment model, with simple application, and is highly cost‐effective as it does not require inputs from relevant laboratory tests. The 2019 WHO CVD Risk Charts provide region‐specific CVD risk prediction models calibrated for 21 distinct Global Burden of Disease (GBD) regions [8]. For the present study, we used the nonlaboratory‐based risk assessment model specifically calibrated for the South Asia region, which encompasses India.

The chart categorizes CVD risk based on the following measures: sex, age, smoking status, systolic blood pressure (SBP), and BMI. Using this WHO chart, the study participants were classified into one of five risk categories: < 5%, 5 to < 10%, 10% to < 20%, 20% to < 30%, and ≥ 30%, wherein, those with ≥ 20% risk were considered at high‐risk of experiencing a CVD event within the next ten years [8].

### 2.5 Operational Definitions

1. Sex: Participants categorized into male or female.

2. Age: Recorded in number of completed years. For the purposes of this study, age groups were stratified as specified by the WHO CVD Risk Chart.

3. Tobacco Smoking Status: classified as “no” or “yes” based on the response to the question “Have you ever smoked tobacco (cigarette, bidi, cigar, hookah, cheroot)”.

4. SBP: Measured in millimeters of mercury (mmHg).

5. BMI: Calculated as weight in kilograms divided by the square of height in meters (kg/m²) and categorized as per the WHO classification. Participants' weight and height were also measured using standardized equipment and procedures.

6. The status of preexisting DM and HTN was determined based on participant response to the following question, “Has any health professional ever diagnosed you with any of the following chronic conditions or diseases” Responses were recorded as either no or yes.

Blood pressure measurements were obtained by trained staff using an Omron HEM 7121 digital monitor. Three consecutive readings were taken with a 1‐min gap, and the average of the second and third measurements was calculated to represent the final value. New cases of HTN were classified as those individuals without a history of diagnosis with HTN observed with an average SBP of 140 mmHg or higher and/or a diastolic blood pressure (DBP) of 90 mmHg or higher, based on the last two readings. Participants were classified as being previously diagnosed hypertensives if they either reported a prior diagnosis of HTN by a healthcare provider or were considered newly diagnosed cases if detected to have high blood pressure during the screening process in LASI.

### 2.6 Covariates

To identify independent predictors of high CVD risk, several sociodemographic and lifestyle variables were extracted based on existing literature.

1. Education was categorized as not educated/up to primary, till secondary school, high school, or college and above.

2. Marital status was classified as never married, currently married/cohabiting, or separated/widowed/others.

3. Work status was dichotomized into currently working and not working.

4. Other sociodemographic variables included religion (Hindu, Muslim, and others), ethnicity/caste (scheduled caste [SC], scheduled tribe [ST], other backward classes [OBC], and others), and place of residence (rural or urban).

5. Household wealth was assessed using the Monthly Per Capita Consumption Expenditure (MPCE), which was divided into quintiles (poorest, poorer, middle, richer, and richest) to serve as a proxy for socioeconomic status.

6. Alcohol use was classified as “no” or “yes” based on whether the participant reported ever consuming alcoholic beverages.

### 2.7 Statistical Analysis

The data were cleaned and analyzed using Stata Version 15.1 (StataCorp, USA). Sample weights provided by LASI were used throughout our analysis using the “svy” command to account for the complex survey design and to ensure the representativeness of the survey results. Categorical variables were reported as frequencies and proportions, and continuous variables as means and standard deviations. Descriptive statistics were reported to stratify the participants as per their CVD risk, separately for males and females, with further subgroup categorization based on the presence or absence of DM or HTN morbidities. Bivariate analyses were conducted to explore unadjusted associations between the participant characteristics and the primary outcome of ≥ 20% CVD risk. To identify independent predictors, an overall multivariable binary logistic regression model was developed. In addition, distinct multivariable logistic regression models were constructed for specific high‐risk subgroups: individuals previously diagnosed with DM, and individuals previously diagnosed with HTN without DM comorbidity. Variables demonstrating a statistically significant association (*p* < 0.05) in the unadjusted logistic regression models were selected for inclusion in the final adjusted models. Since age, sex, smoking status, SBP, and BMI constitute the WHO CVD Risk chart, they were excluded as covariates from the regression models. The results were expressed as odds ratios (ORs) with 95% confidence intervals (CIs). The Hosmer–Lemeshow test was conducted to check for model fit, but interpreted with caution due to its known hypersensitivity in large survey samples. Model discrimination was evaluated using the area under the receiver operating characteristic curve (AUC‐ROC), and model calibration was visually assessed using a calibration plot comparing predicted probabilities against observed frequencies. A value of *p* < 0.05 was considered statistically significant throughout the analysis.

### 2.8 Ethical Considerations

The ethics approval for the LASI was granted by the Indian Council of Medical Research (ICMR) and the International Institute for Population Sciences. The data from LASI Wave‐1 is public and anonymized; no separate ethical clearance was required to conduct this analysis.

## 3 Results

Prevalence of self‐reported DM among females was 11.84% (95% CI: 11.00, 12.73) and 12.33% (95% CI: 11.30, 13.44) in males, whereas the prevalence of HTN among females was 47.20% (95% CI: 45.91, 48.49) and 45.21% (95% CI: 43.82, 46.61) in males. The DM prevalence increased with age, particularly in the 50–54 age group. Both DM and HTN were higher among individuals with higher education levels, urban residents, those not working, and those with a BMI of ≥ 35 kg/m². Lifestyle factors like smoking and alcohol usage also influenced both the DM and HTN prevalence (Tables S1 and S2).

The weighted prevalence of individuals with high 10‐year risk of CVD ( ≥ 20%) in the study was 2.67% (95% CI: 2.44, 2.91), ≥ 10% to < 20% was 28.41% (95% CI: 27.60, 29.25), and overall ≥ 10% risk was 31.08% (95% CI: 30.25, 31.93). The weighted prevalence of individuals with a 10‐year risk of CVD of ≥ 5% and < 10% in the study was 35.97% (95% CI: 35.11, 36.84).

Table 1 reports the distribution of sociodemographic and lifestyle factors and their association with the outcome of CVD risk of ≥ 20%. On bivariate analysis, education level, marital status, work status, alcohol use, and previous diagnosis of DM and HTN had a statistically significant association with high risk of CVD. On adjusted analysis, significantly higher odds of being at a ≥ 20% risk of CVD were observed in individuals with marital status as separated/widowed/others (adjusted odds ratio [aOR] = 3.80, 95% CI: 1.17, 12.32), alcohol usage (aOR = 2.66, 95% CI: 2.08, 3.40), and previous diagnosis of HTN (aOR = 1.71, 95% CI: 1.34, 2.19). Significantly lower odds of being at a ≥ 20% risk of CVD was observed among individuals with high school education (aOR = 0.65, 95% CI: 0.43, 0.99), college and above education (aOR = 0.50, 95% CI: 0.33, 0.75), and work status as currently working (aOR = 0.20, 95% CI: 0.15, 0.25). The model demonstrated good discrimination with an AUC of 0.77. Furthermore, visual inspection of the calibration plot indicated strong agreement between the predicted and observed probabilities of high CVD risk, confirming adequate model calibration (Figure S1).

**Table 1:** Distribution of the study population with their CVD risk (binary logistic regression to compare ≥ 20% vs. < 20% risk).

Characteristics	CVD Risk					Unadjusted OR [95% CI]	Adjusted OR [95% CI]
	< 5% (*n* = 19,595) *n* (%)	5 to < 10% (*n* = 21,175) *n* (%)	10 to < 20% (*n* = 16,103) *n* (%)	20 to < 30% (*n* = 1559) *n* (%)	≥ 30% (*n* = 46) *n* (%)		
**Highest education**							
Not educated/up to primary	4632 (32.43)	5292 (35.05)	4279 (29.47)	427 (2.99)	13 (0.06)	Ref	Ref
Till secondary school	4308 (34.59)	4046 (34.54)	2860 (28.22)	290 (2.6)	12 (0.05)	0.87 [0.67, 1.11]	0.96 [0.74, 1.26]
High school	1145 (45.45)	870 (32.18)	592 (20.65)	58 (1.72)	0 (0)	0.56 [0.36, 0.85]*	0.65 [0.43, 0.99]*
College and above	1286 (38.16)	1077 (36.08)	821 (24.2)	56 (1.44)	2 (0.12)	0.51 [0.34, 0.76]*	0.50 [0.33, 0.75]*
**Marital status**							
Never married	241 (51.83)	265 (28.36)	162 (18.18)	13 (1.63)	0 (0)	Ref	Ref
Currently married/cohabiting	17,335 (36.79)	16,934 (36.24)	11,549 (24.72)	1054 (2.19)	34 (0.05)	1.38 [0.51, 3.72]	2.33 [0.74, 7.32]
Separated/widowed/others	2018 (16.34)	3975 (35.29)	4392 (43.89)	492 (4.4)	12 (0.08)	2.83 [1.05, 7.66]*	3.80 [1.17, 12.32]*
**Religion**							
Hindu	14,626 (32.98)	15,479 (36.21)	11,612 (28.21)	1096 (2.56)	22 (0.04)	Ref	—
Muslim	2219 (31.99)	2460 (35.26)	2042 (29.72)	245 (2.91)	9 (0.12)	1.17 [0.94, 1.46]	
Others	2747 (34.23)	3235 (34.12)	2449 (28.73)	218 (2.84)	15 (0.09)	1.13 [0.80, 1.61]	
**Ethnicity**							
SC	3357 (33.3)	3535 (35.5)	2708 (28.36)	270 (2.81)	6 (0.03)	Ref	—
ST	3270 (31.36)	4016 (39.08)	2842 (26.82)	319 (2.66)	15 (0.08)	0.96 [0.73, 1.27]	
OBC	7748 (33.84)	7935 (35.76)	5999 (27.97)	518 (2.37)	13 (0.06)	0.85 [0.67, 1.07]	
Other	4535 (31.85)	4963 (35.67)	3970 (29.56)	400 (2.85)	11 (0.07)	1.03 [0.82, 1.29]	
**MPCE quintile**							
Poorest	3646 (32.44)	4240 (35.95)	3257 (28.76)	339 (2.83)	5 (0.03)	Ref	—
Poorer	3763 (31.56)	4394 (37.83)	3296 (27.95)	327 (2.61)	7 (0.05)	0.93 [0.74, 1.16]	
Middle	4009 (33.6)	4146 (35.01)	3319 (28.44)	308 (2.89)	9 (0.06)	1.03 [0.76, 1.40]	
Richer	4055 (33.51)	4225 (34.16)	3199 (29.81)	316 (2.41)	17 (0.11)	0.88 [0.70, 1.11]	
Richest	4122 (33.81)	4170 (36.9)	3032 (26.99)	269 (2.26)	8 (0.03)	0.80 [0.61, 1.05]	
**Residence**							
Rural	12,472 (32.11)	13,973 (36.26)	10,594 (28.87)	1073 (2.71)	30 (0.06)	Ref	—
Urban	7123 (34.84)	7202 (35.32)	5509 (27.39)	486 (2.4)	16 (0.05)	0.89 [0.70, 1.13]	
**Work status**							
Not working	1983 (15.42)	3865 (30.61)	5927 (46.6)	902 (7.2)	31 (0.18)	Ref	Ref
Currently working	10,305 (35.08)	11,362 (39.69)	6639 (23.75)	438 (1.46)	11 (0.03)	0.19 [0.16, 0.23]**	0.20 [0.15, 0.25]**
**Alcohol use**							
No	17,976 (35.99)	17,207 (35.54)	12,146 (26.44)	1048 (2)	20 (0.03)	Ref	Ref
Yes	1618 (15.12)	3967 (38.48)	3954 (40.01)	511 (6.22)	26 (0.17)	3.29 [2.66, 4.07]**	2.66 [2.08, 3.40]**
**Previously diagnosed DM**							
No	18,051 (34.63)	18,506 (35.99)	13,461 (26.83)	1292 (2.51)	36 (0.05)	Ref	Ref
Yes	1539 (19.91)	2662 (35.83)	2638 (40.73)	267 (3.42)	10 (0.11)	1.39 [1.12, 1.74]*	1.21 [0.90, 1.62]
**Previously diagnosed HTN**							
No	15,854 (36.34)	15,436 (36.22)	10,438 (25.33)	870 (2.09)	21 (0.02)	Ref	Ref
Yes	3737 (23.09)	5733 (35.25)	5661 (37.38)	689 (4.13)	25 (0.15)	2.06 [1.70, 2.50]**	1.71 [1.34, 2.19]**

Abbreviations: AUC‐ROC, area under the receiver operating characteristic curve; BMI, body mass index; CI, confidence interval; CVD, cardiovascular disease; DM, diabetes mellitus; HTN, hypertension; MPCE, monthly per capita expenditure; OBC, other backward classes; OR, odds ratio; Ref, reference; SC, scheduled caste; ST, scheduled tribe.

* *p* < 0.05

** *p* < 0.001.

*p*‐value for Hosmer–Lemeshow test = 0.50, AUC‐ROC = 0.77.

Table 2 reports the distribution of sociodemographic and lifestyle factors associated with CVD risk of ≥ 20% among those previously diagnosed with DM (*n* = 7843). In this subset of participants with DM, the weighted prevalence of individuals with high risk of CVD ( ≥ 20%) was 3.53% (95% CI: 2.91, 4.27). On adjusted analysis, significantly higher odds of being at a ≥ 20% risk of CVD were observed in individuals with alcohol usage (aOR = 3.13, 95% CI: 2.01, 4.89). Significantly lower odds of being at a ≥ 20% risk of CVD were observed among individuals with marital status as never married (aOR = 0.05, 95% CI: 0.01, 0.44), and work status as currently working (aOR = 0.24, 95% CI: 0.14, 0.38). The model demonstrated good discrimination with an AUC of 0.74 (Figure S2).

**Table 2:** Predictors of high CVD risk ( ≥ 20% risk) in people with DM (*n* = 7843).

Characteristics	Unadjusted OR [95% CI]	Adjusted OR [95% CI]
**Highest education**		
Not educated/up to primary	Ref	—
Till secondary school	0.68 [0.39, 1.17]	
High school	0.73 [0.33, 1.62]	
College and above	0.52 [0.25, 1.08]	
**Marital status**		
Currently married/cohabiting	Ref	Ref
Never married	0.06 [0.01, 0.44]*	0.05 [0.01, 0.44]*
Separated/widowed/others	1.28 [0.77, 2.12]	1.53 [0.94, 2.47]
**Religion**		
Hindu	Ref	Ref
Muslim	1.31 [0.79, 2.15]	1.62 [0.92, 2.86]
Others	2.24 [1.12, 4.50]*	1.77 [0.85, 3.66]
**Ethnicity**		
SC	Ref	—
ST	1.26 [0.54, 2.95]	
OBC	0.69 [0.36, 1.34]	
Other	0.98 [0.51, 1.88]	
**MPCE quintile**		
Poorest	Ref	—
Poorer	1.32 [0.67, 2.61]	
Middle	1.13 [0.54, 2.35]	
Richer	0.90 [0.45, 1.82]	
Richest	0.83 [0.41, 1.68]	
**Residence**		
Rural	Ref	—
Urban	0.80 [0.54, 1.18]	
**Work status**		
Not working	Ref	Ref
Currently working	0.23 [0.14, 0.37]**	0.24 [0.14, 0.38]**
**Alcohol use**		
No	Ref	Ref
Yes	4.05 [2.71, 6.05]**	3.13 [2.01, 4.89]**
**Previously diagnosed HTN**		
No	Ref	—
Yes	1.52 [0.99, 2.34]	

Abbreviations: AUC‐ROC, area under the receiver operating characteristic curve; CI, confidence interval; CVD, cardiovascular disease; DM, diabetes mellitus; HTN, hypertension; MPCE, monthly per capita expenditure; OBC, other backward classes; OR, odds ratio; Ref, reference; SC, scheduled caste; ST, scheduled tribe.

* *p* < 0.05

** *p* < 0.001.

*p*‐value for Hosmer–Lemeshow test = 0.98, AUC‐ROC = 0.74.

Table 3 reports the distribution of sociodemographic and lifestyle factors associated with CVD risk of ≥ 20% among those previously diagnosed with HTN without DM comorbidity (*n* = 12,764). In this subset of participants with HTN, the weighted prevalence of individuals with high risk of CVD ( ≥ 20%) was found to be 4.36% (95% CI: 3.60, 5.27). On adjusted analysis, significantly higher odds of being at a ≥ 20% risk of CVD were observed in individuals with alcohol usage (aOR = 3.08, 95% CI: 2.07, 4.58). Significantly lower odds of being at a ≥ 20% risk of CVD was observed among individuals with college and above education (aOR = 0.47, 95% CI: 0.24, 0.91), marital status as never married (aOR = 0.09, 95% CI: 0.02, 0.47), and work status as currently working (aOR = 0.30, 95% CI: 0.20, 0.44). The model demonstrated good discrimination with an AUC of 0.73 (Figure S3).

**Table 3:** Predictors of high CVD risk (≥ 20% risk) in people with HTN without DM comorbidity (*n* = 12,764).

Characteristics	Unadjusted OR [95% CI]	Adjusted OR [95% CI]
**Highest education**		
Not educated/up to primary	Ref	Ref
Till secondary school	1.05 [0.67, 1.66]	1.09 [0.65, 1.83]
High school	0.72 [0.38, 1.40]	0.77 [0.37, 1.59]
College and above	0.51 [0.27, 0.95]*	0.47 [0.24, 0.91]*
**Marital status**		
Currently married/cohabiting	Ref	Ref
Never married	0.07 [0.02, 0.28]**	0.09 [0.02, 0.47]*
Separated/widowed/others	1.41 [0.97, 2.05]	1.45 [0.77, 2.71]
**Religion**		
Hindu	Ref	—
Muslim	0.81 [0.54, 1.22]	
Others	0.76 [0.45, 1.29]	
**Ethnicity**		
SC	Ref	—
ST	1.19 [0.73, 1.95]	
OBC	1.26 [0.77, 2.06]	
Other	1.28 [0.86, 1.92]	
**MPCE quintile**		
Poorest	Ref	Ref
Poorer	0.47 [0.31, 0.74]*	0.56 [0.26, 1.20]
Middle	1.00 [0.52, 1.92]	0.65 [0.31, 1.39]
Richer	0.55 [0.36, 0.84]*	0.68 [0.32, 1.43]
Richest	0.53 [0.34, 0.83]*	0.80 [0.38, 1.67]
**Residence**		
Rural	Ref	—
Urban	1.04 [0.63, 1.72]	
**Work status**		
Not working	Ref	Ref
Currently working	0.24 [0.17, 0.34]**	0.30 [0.20, 0.44]**
**Alcohol use**		
No	Ref	Ref
Yes	5.38 [3.34, 8.66]**	3.08 [2.07, 4.58]**

Abbreviations: AUC‐ROC, area under the receiver operating characteristic curve; CI, confidence interval; CVD, cardiovascular disease; DM, diabetes mellitus; HTN, hypertension; MPCE, monthly per capita expenditure; OBC, other backward classes; OR, odds ratio; Ref, reference; SC, scheduled caste; ST, scheduled tribe.

* *p* < 0.05

** *p* < 0.001.

*p*‐value for Hosmer–Lemeshow test = 0.97, AUC‐ROC = 0.73.

Individuals with a history of HTN or DM, those who consume alcohol, and those who are married or widowed exhibited significantly higher odds of a high CVD risk ( ≥ 10% risk). Conversely, higher educational attainment and current employment were associated with significantly lower odds of high risk (Table S3).

The treatment status of participants with either DM or HTN comorbidity was assessed after stratification with their CVD risk status. Regarding anti‐DM treatment, in the highest risk category ( ≥ 30%), 91.81% of individuals received treatment, however, the proportion of individuals receiving treatment was significantly lower in the lower risk categories (20%–30% risk group: 80.83%; 10%–20% risk group: 83.45%; 5%–10% risk group: 79.53%; < 5% risk group: 79.8%). Regarding antihypertensive treatment, in the ≥ 30% risk category, only 79.18% were currently taking treatment, 20%–30% risk group: 73.67%; 10%–20% risk group: 69.64%; 5%–10% risk group: 78.01%; > 5% risk group: 56.53%.

**Figure 1:**
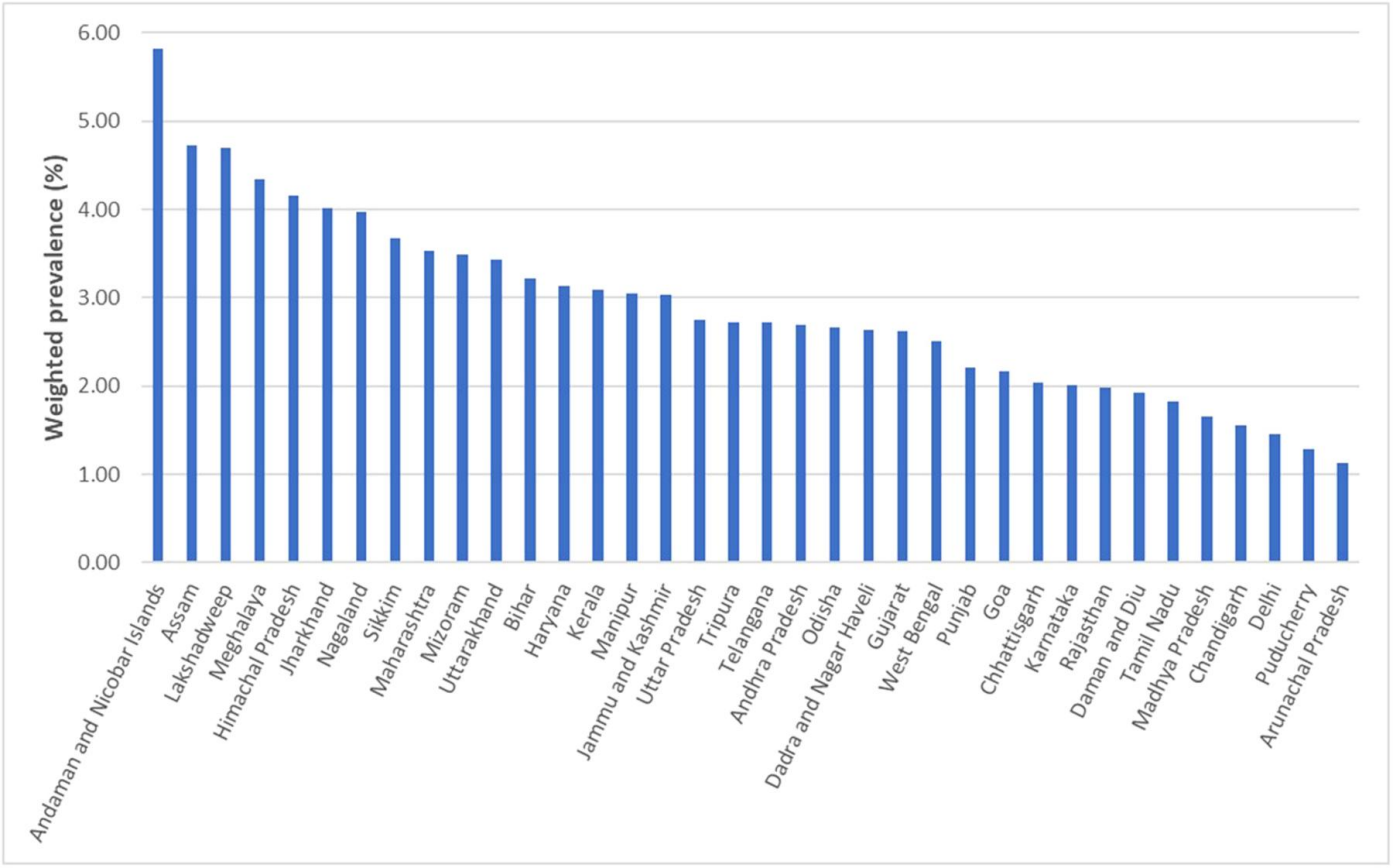
Weighted prevalence of individuals at high risk ( ≥ 20%) for CVD across states and union territories of India. CVD, cardiovascular disease.

The geographic distribution of CVD risk across India was mapped (Figure 1). The lowest prevalence of individuals at high risk ( ≥ 20%) for CVD was found in one of the northeastern study regions (1.13%), Puducherry (1.28%) and Delhi (1.46%), while the highest prevalence was found in Andaman and Nicobar Islands (5.82%), northeastern state of Assam (4.73%) and the islands of Lakshadweep (4.70%).

## 4 Discussion

The present study investigated the CVD risk burden in a middle‐aged and geriatric population cohort in India. The prevalence of high risk of CVD ( ≥ 20%) observed in this study (2.67%) is several‐folds higher compared with estimates from the National Family Health survey fourth round (2015–2016) (0.03%) [13], likely attributable to the difference in age‐structure of the study populations. The NFHS‐4 study assessed participants in the middle‐aged group (40–54 years), whereas this LASI study comprised the middle‐aged and geriatric age‐group with higher burden of comorbidities, especially HTN and DM, thereby predisposing to the high risk of CVD ( ≥ 20%).

Previous studies have observed a significant association between high lifetime risk of CVDs and a history of DM or HTN [13, 14, 15]. Similarly, in the present study, the prevalence of high CVD risk among patients with DM and HTN was significantly higher compared to the overall study sample, indicative of the need for personalized treatment and enhanced focus in this vulnerable population.

This study demonstrated a significant association between lower CVD risk ( ≥ 20%) and higher educational attainment, which may reflect the role of awareness and health literacy on cardiovascular health, also indicated in previous cohort studies, which reported an inverse correlation of lifetime CVD risk with educational attainment [16]. Furthermore, our findings indicate that separated or widowed individuals have significantly higher odds of CVD risk compared to currently married individuals, corroborating prior evidence suggestive of the efficacy of spousal support in facilitating healthier lifestyle choices and mitigating stress, factors crucial for minimizing the risk of developing cardiovascular disease [17].

Interestingly, while our descriptive data indicated variations in elevated CVD risk across religious and ethnic categories, these variables did not emerge as statistically significant independent predictors in the multivariable models. This attenuation could suggest that the influence of caste and religion on CVD risk is heavily mediated by downstream socioeconomic factors, such as educational attainment, wealth, and employment status, which remained strong independent predictors in our analysis. Addressing cardiovascular disparities in India, therefore, requires intersectional public health policies that do not merely look at clinical risk but actively dismantle the socio‐economic intermediaries that drive higher disease burdens among historically marginalized communities.

In this study, patients with DM or HTN had more than three times higher odds of belonging to the high‐risk CVD group. Previous studies have demonstrated a dose‐dependent association of alcohol intake with varying intensity of cardiovascular risk, with a likely additive effect on the risk of incidence of CVDs [18, 19]. Another study reported a likely causal association between alcohol consumption and the risk of CVD, especially above an alcohol intake of 12 g/d [20].

Work‐related stressors such as job strain, shift work, and long working hours, have been linked with higher risk of CVD [21, 22]. However, in the present study, those currently working had significantly lower odds of having high CVD risk. Due to the cross‐sectional nature of our analysis, this inverse association should not be interpreted as employment actively reducing cardiovascular risk. Rather, it is highly indicative of the “healthy worker effect” [23] and reverse causation. In cross‐sectional studies, individuals with declining health often exit the workforce prematurely, while those who remain employed represent a preselected, healthier group. Therefore, current employment status in this analysis likely serves as a proxy marker for baseline physical capacity rather than a protective exposure against CVD. A previous study from Ethiopia among persons with HTN had also indicated a higher risk of CVD in those unemployed [24].

A meta‐analysis of randomized controlled trials of BP‐lowering treatment in patients with DM ascertained that for each 10‐mmHg lowering of the SBP, the risk for adverse cardiovascular outcomes is significantly lowered [25]. Furthermore, the benefits of strict glycemic control on long‐term cardiovascular health are well‐established from large clinical trials [26]. Consequently, ensuring universal standard care for patients with HTN and DM warrants high prioritization worldwide [27]. However, in the present study, a substantial proportion of patients with DM or HTN were not currently receiving treatment, even among those in the highest risk groups. Although basic antidiabetic and antihypertensive medications are nominally provided free of charge in Indian public health facilities, true accessibility is frequently hindered by other health‐system‐related barriers. These challenges include frequent supply‐chain stock‐outs, the limited pharmacological scope of the basic treatments available, over‐burdened healthcare infrastructure, and hidden out‐of‐pocket costs [28]. Therefore, effectively mitigating this CVD burden requires comprehensive health‐system strengthening to ensure consistent drug availability and adequate facility support, rather than solely focusing on patient‐level awareness and adherence.

Our study does have certain limitations. First, we lacked longitudinal follow‐up data, and consequently, we can only estimate the predicted future risk. Moreover, we cannot establish causal relationships between the identified sociodemographic determinants and high CVD risk. Second, although our total sample size is large, only a small fraction of participants (2.74%, or *n* = 1605) comprised the high‐risk ( ≥ 20%) group. This imbalance may introduce instability into our multivariable logistic regression estimates, so any results with wide 95% CIs should be interpreted with caution. Third, because the 40–44 years age subgroup in the LASI dataset exclusively comprises spouses of older primary respondents, findings for this specific age bracket may not be entirely generalizable to the overall 40‐ to 44‐year‐old population in India, despite the application of survey weights. Fourth, the WHO CVD Risk Chart optimally calculates risk using current smoking status; however, our study used “ever smoked tobacco” as a proxy variable. This methodological choice was necessitated by the survey's skip‐logic and massive missing data for the current smoking variable. Although the approach ensured maximum sample retention for our models, it may lead to a slight overestimation of the calculated CVD risk for former smokers who have abstained for prolonged periods. Fifth, because participants on antihypertensive medication were also included in the cohort, their therapeutically lowered SBP was used in the WHO chart calculations, which likely leads to an underestimation of their true underlying CVD risk. The LASI survey involved self‐reported questions pertaining to CVD‐risk, therefore, chances of recall bias are present while self‐reporting of sensitive behaviors can be influenced by social desirability bias [29]. DM status was self‐reported in this analysis, which could be an underestimation as a substantial proportion of patients with DM remain undiagnosed in India [30]. Despite these limitations, the present study has its own strengths. The huge representative sample of the survey ensures generalizability to the Indian population, while the application of the standardized and validated methodology of the WHO CVD risk stratification ensured consistency and reliability of the study findings.

## 5 Conclusion

More than 3 in 100 patients with DM and HTN in India aged ≥ 40 years have a ≥ 20% risk of a 10‐year CVD event, indicating an urgent need for primary care‐focused targeted interventions. To address this burden, the WHO nonlaboratory‐based risk assessment model could be routinely integrated into clinical practice and actively used by frontline health workers. Deploying this cost‐effective tool at the community level will significantly enhance patient awareness of their CVD vulnerability, helping them to take timely preventive measures. Stopping the harmful use of alcohol, lowering blood pressure in HTN cases, and improving glycemic control through universal standard care coverage warrant high prioritization in the country to effectively lower CVD and associated complications, particularly in the high‐risk vulnerable groups.

## Author Contributions

**Vansh Maheshwari:** methodology, formal analysis, writing – original draft preparation, writing – reviewing and editing. **Shyamashree Das:** methodology, writing – original draft preparation, writing – reviewing and editing. **Saurav Basu:** conceptualization, visualization, writing – reviewing and editing, supervision. All authors read and approved the final version of the manuscript.

## Funding

The authors have nothing to report.

## Ethics Statement

Use of large language models, AI and machine learning tools: AI tool (ChatGPT‐4) was only used for language checking of the manuscript.

## Conflicts of Interest

The authors declare no conflicts of interest.

## Supporting information

Supporting file

## Data Availability

LASI datasets are freely available to use upon a reasonable request to IIPS (https://www.iipsdata.ac.in/datacatalog_detail/5). The data that support the findings of this study are openly available in Longitudinal Ageing Study in India (LASI) at https://www.iipsindia.ac.in/lasi.
