# Supplementary material for: Cardiovascular Risk Assessment and Its Determinants Among Older Adults in India: Evidence From a Nationally Representative Survey
Source: Chronic Dis Transl Med. 2026 May 3;12(3):209–18. doi: 10.1002/cdt3.70052 (PMC13394149; doi:10.1002/cdt3.70052)
Supplement: Supplementary file 1 [file CDT3-12-209-s001.docx]

**SUPPLEMENTARY MATERIAL**

**Table S1: Distribution of sociodemographic characteristics stratified by DM status**

| **Characteristics** | **Males (n = 27792)** | | **Females (n = 36474)** | | **Total (N = 64266)** | |
| --- | --- | --- | --- | --- | --- | --- |
|  | **Without DM**  **(n = 24076)** | **With DM**  **(3623)** | **Without DM**  **(n = 32178)** | **With DM**  **(n = 4220)** | **Without DM**  **(n = 56254)** | **With DM**  **(n = 7843)** |
| **Age (years)** |  |  |  |  |  |  |
| 40-44 | 66 (96.52) | 5 (3.479) | 4247 (96.28) | 214 (3.72) | 4313 (96.28) | 219 (3.72) |
| 45-49 | 5547 (93.31) | 477 (6.686) | 6835 (91.69) | 547 (8.314) | 12382 (92.38) | 1024 (7.62) |
| 50-54 | 4498 (87.04) | 583 (12.96) | 5320 (90.52) | 656 (9.477) | 9818 (88.89) | 1239 (11.11) |
| 55-59 | 3971 (88.47) | 576 (11.53) | 4805 (85.44) | 791 (14.56) | 8776 (86.77) | 1367 (13.23) |
| 60-64 | 3967 (86.05) | 720 (13.95) | 4710 (86.55) | 843 (13.45) | 8677 (86.32) | 1563 (13.68) |
| 65-69 | 3626 (84.41) | 743 (15.59) | 3856 (84.83) | 701 (15.17) | 7482 (84.63) | 1444 (15.37) |
| 70-74 | 2401 (84.85) | 519 (15.15) | 2405 (82.29) | 468 (17.71) | 4806 (83.51) | 987 (16.49) |
| **Highest Education** |  |  |  |  |  |  |
| Not educated/ Up to Primary | 6941 (88.92) | 966 (11.08) | 6708 (84.92) | 1171 (15.08) | 13649 (86.95) | 2137 (13.05) |
| Till Secondary School | 6099 (85.08) | 1165 (14.92) | 4506 (80.03) | 922 (19.97) | 10605 (82.98) | 2087 (17.02) |
| High School | 1468 (81.66) | 374 (18.34) | 976 (89.41) | 182 (10.59) | 2444 (84.75) | 556 (15.25) |
| College and above | 1879 (78.29) | 551 (21.71) | 1118 (75.7) | 214 (24.3) | 2997 (77.37) | 765 (22.63) |
| **Marital status** |  |  |  |  |  |  |
| Never Married | 418 (94.12) | 26 (5.88) | 331 (95.42) | 37 (4.58) | 749 (94.74) | 63 (5.26) |
| Currently Married/Cohabiting | 21717 (87.35) | 3356 (12.65) | 23386 (89.31) | 2897 (10.69) | 45103 (88.36) | 6253 (11.64) |
| Separated/Widowed/Others | 1939 (89.98) | 241 (10.02) | 8460 (86.3) | 1286 (13.7) | 10399 (86.97) | 1527 (13.03) |
| **Work Status** |  |  |  |  |  |  |
| Not working | 5542 (79.3) | 1491 (20.7) | 5923 (87.43) | 949 (12.57) | 11465 (83.54) | 2440 (16.46) |
| Currently Working | 17430 (90.62) | 1967 (9.38) | 11407 (93.84) | 685 (6.16) | 28837 (91.89) | 2652 (8.11) |
| **Religion** |  |  |  |  |  |  |
| Hindu | 17754 (88.49) | 2621 (11.51) | 23730 (89.2) | 2870 (10.8) | 41484 (88.9) | 5491 (11.1) |
| Muslim | 2693 (82.84) | 496 (17.16) | 3714 (83.85) | 717 (16.15) | 6407 (83.42) | 1213 (16.58) |
| Others | 3627 (85.69) | 506 (14.31) | 4731 (88.39) | 633 (11.61) | 8358 (87.3) | 1139 (12.7) |
| **Ethnicity** |  |  |  |  |  |  |
| SC | 4127 (91.24) | 467 (8.76) | 5555 (91.81) | 588 (8.19) | 9682 (91.57) | 1055 (8.43) |
| ST | 4557 (94.95) | 379 (5.05) | 5969 (95.62) | 432 (4.38) | 10526 (95.33) | 811 (4.67) |
| OBC | 9046 (86.06) | 1510 (13.94) | 12078 (86.71) | 1679 (13.29) | 21124 (86.43) | 3189 (13.57) |
| Other | 5542 (85.29) | 1107 (14.71) | 7469 (86.94) | 1326 (13.06) | 13011 (86.23) | 2433 (13.77) |
| **MPCE Quintile** |  |  |  |  |  |  |
| Poorest | 4983 (91.93) | 434 (8.07) | 6503 (92.46) | 599 (7.54) | 11486 (92.24) | 1033 (7.76) |
| Poorer | 4900 (89.5) | 622 (10.5) | 6671 (92.2) | 684 (7.8) | 11571 (91.04) | 1306 (8.96) |
| Middle | 4860 (88.68) | 673 (11.32) | 6481 (89.64) | 843 (10.36) | 11341 (89.24) | 1516 (10.76) |
| Richer | 4792 (85.84) | 841 (14.16) | 6378 (86.35) | 966 (13.65) | 11170 (86.14) | 1807 (13.86) |
| Richest | 4541 (81.7) | 1053 (18.3) | 6145 (80.83) | 1128 (19.17) | 10686 (81.21) | 2181 (18.79) |
| **Residence** |  |  |  |  |  |  |
| Rural | 16450 (91.51) | 1616 (8.49) | 21503 (92.84) | 1776 (7.16) | 37953 (92.26) | 3392 (7.74) |
| Urban | 7626 (78.86) | 2007 (21.14) | 10675 (79.78) | 2444 (20.22) | 18301 (79.41) | 4451 (20.59) |
| **BMI (kg/m2)** |  |  |  |  |  |  |
| <20 | 7169 (95.25) | 399 (4.75) | 8395 (96.39) | 324 (3.61) | 15564 (95.86) | 723 (4.14) |
| 20-24 | 9701 (87.79) | 1412 (12.21) | 11366 (90.83) | 1257 (9.17) | 21067 (89.42) | 2669 (10.58) |
| 25-29 | 4091 (79.76) | 1128 (20.24) | 6878 (82.33) | 1454 (17.67) | 10969 (81.38) | 2582 (18.62) |
| 30-35 | 694 (69.24) | 235 (30.76) | 2158 (79.03) | 633 (20.97) | 2852 (76.83) | 868 (23.17) |
| ≥35 | 117 (80.08) | 40 (19.92) | 601 (63.36) | 183 (36.64) | 718 (65.48) | 223 (34.52) |
| **Smoking Status** |  |  |  |  |  |  |
| No | 10098 (83.07) | 2089 (16.93) | 25792 (87.59) | 3677 (12.41) | 35890 (86.37) | 5766 (13.63) |
| Yes | 13796 (91.03) | 1499 (8.97) | 6210 (92.86) | 514 (7.14) | 20006 (91.57) | 2013 (8.43) |
| **Alcohol Use** |  |  |  |  |  |  |
| No | 15405 (86.68) | 2533 (13.32) | 30637 (88.35) | 4127 (11.65) | 46042 (87.78) | 6660 (12.22) |
| Yes | 8495 (90.07) | 1059 (9.93) | 1370 (95.09) | 68 (4.91) | 9865 (90.56) | 1127 (9.44) |
| **Previously diagnosed HTN** |  |  |  |  |  |  |
| No | 19508 (93.32) | 1595 (6.68) | 23981 (94.62) | 1546 (5.38) | 43489 (94.04) | 3141 (5.96) |
| Yes | 4568 (68.25) | 2028 (31.75) | 8196 (73.26) | 2674 (26.74) | 12764 (71.42) | 4702 (28.58) |
| **Mean (SD) SBP in mmHg** | 126.25 (18.36) | 133.54 (18.71) | 125.36 (18.66) | 133.20 (18.29 | 125.73 (18.54) | 133.35 (18.47) |

Abbreviations: DM = diabetes; SC = scheduled caste; ST = scheduled tribe; OBC = other backward classes; MPCE = monthly per capita expenditure; BMI = body mass index; HTN = hypertension; SD = standard deviation; SBP = systolic blood pressure

**Table S2: Distribution of sociodemographic characteristics stratified by HTN status (both old and new cases)**

| **Characteristics** | **Males (n = 27792)** | | **Females (n = 36474)** | | **Total (N = 64266)** | |
| --- | --- | --- | --- | --- | --- | --- |
|  | **Without HTN**  **(n = 13421)** | **With HTN**  **(n = 12432)** | **Without HTN**  **(n = 17582)** | **With HTN**  **(n = 16734)** | **Without HTN**  **(n = 31003)** | **With HTN**  **(n = 29166)** |
| **Age (years)** |  |  |  |  |  |  |
| 40-44 | 37 (60.27) | 29 (39.73) | 2833 (70.06) | 1310 (29.94) | 2870 (70.01) | 1339 (29.99) |
| 45-49 | 3336 (63.87) | 2210 (36.13) | 4349 (64.07) | 2580 (35.93) | 7685 (63.99) | 4790 (36.01) |
| 50-54 | 2669 (56.08) | 2028 (43.92) | 3003 (54.14) | 2629 (45.86) | 5672 (55.05) | 4657 (44.95) |
| 55-59 | 2218 (57.53) | 2008 (42.47) | 2570 (51.62) | 2724 (48.38) | 4788 (54.2) | 4732 (45.8) |
| 60-64 | 2154 (52.06) | 2262 (47.94) | 2246 (45.59) | 3034 (54.41) | 4400 (48.58) | 5296 (51.42) |
| 65-69 | 1843 (47.85) | 2291 (52.15) | 1650 (40.68) | 2662 (59.32) | 3493 (44.05) | 4953 (55.95) |
| 70-74 | 1164 (47.48) | 1604 (52.52) | 931 (36.91) | 1795 (63.09) | 2095 (41.94) | 3399 (58.06) |
| **Highest Education** |  |  |  |  |  |  |
| Not educated/ Up to Primary | 3973 (55.32) | 3483 (44.68) | 3632 (50.09) | 3914 (49.91) | 7605 (52.74) | 7397 (47.26) |
| Till Secondary School | 3292 (52.66) | 3504 (47.34) | 2530 (47.68) | 2571 (52.32) | 5822 (50.59) | 6075 (49.41) |
| High School | 778 (53.66) | 934 (46.34) | 542 (50.53) | 526 (49.47) | 1320 (52.42) | 1460 (47.58) |
| College and above | 953 (40.27) | 1284 (59.73) | 635 (48.22) | 565 (51.78) | 1588 (42.99) | 1849 (57.01) |
| **Marital status** |  |  |  |  |  |  |
| Never Married | 224 (61.96) | 157 (38.04) | 156 (69.62) | 171 (30.38) | 380 (65.8) | 328 (34.2) |
| Currently Married/Cohabiting | 12227 (55.11) | 11220 (44.89) | 13640 (56.66) | 11126 (43.34) | 25867 (55.91) | 22346 (44.09) |
| Separated/Widowed/Others | 970 (50.29) | 1054 (49.71) | 3785 (42.38) | 5437 (57.62) | 4755 (43.82) | 6491 (56.18) |
| **Work Status** |  |  |  |  |  |  |
| Not working | 2747 (42.61) | 3939 (57.39) | 2928 (46.76) | 3563 (53.24) | 5675 (44.76) | 7502 (55.24) |
| Currently Working | 10172 (59.31) | 7863 (40.69) | 6851 (60.97) | 4479 (39.03) | 17023 (59.96) | 12342 (40.04) |
| **Religion** |  |  |  |  |  |  |
| Hindu | 10108 (56.11) | 8888 (43.89) | 13308 (54.29) | 11718 (45.71) | 23416 (44.94) | 20606 (100) |
| Muslim | 1526 (50.04) | 1454 (49.96) | 1840 (44.27) | 2391 (55.73) | 3366 (46.69) | 3845 (53.31) |
| Others | 1787 (46.11) | 2089 (53.89) | 2433 (49.66) | 2623 (50.34) | 4220 (48.21) | 4712 (51.79) |
| **Ethnicity** |  |  |  |  |  |  |
| SC | 2369 (58.82) | 1934 (41.18) | 3116 (55.87) | 2715 (44.13) | 5485 (57.11) | 4649 (42.89) |
| ST | 2430 (60.44) | 2204 (39.56) | 3257 (60.92) | 2748 (39.08) | 5687 (60.71) | 4952 (39.29) |
| OBC | 5229 (53.75) | 4617 (46.25) | 6854 (53.39) | 6129 (46.61) | 12083 (53.54) | 10746 (46.46) |
| Other | 2963 (51.9) | 3201 (48.1) | 3805 (47.41) | 4465 (52.59) | 6768 (49.33) | 7666 (50.67) |
| **MPCE Quintile** |  |  |  |  |  |  |
| Poorest | 2876 (61.19) | 2177 (38.81) | 3712 (57.67) | 2965 (42.33) | 6588 (59.17) | 5142 (40.83) |
| Poorer | 2840 (57.22) | 2320 (42.78) | 3695 (55.23) | 3238 (44.77) | 6535 (56.08) | 5558 (43.92) |
| Middle | 2722 (54.62) | 2467 (45.38) | 3535 (52.69) | 3378 (47.31) | 6257 (53.5) | 5845 (46.5) |
| Richer | 2590 (53.19) | 2660 (46.81) | 3404 (50.79) | 3533 (49.21) | 5994 (51.78) | 6193 (48.22) |
| Richest | 2393 (46.75) | 2808 (53.25) | 3236 (46.75) | 3620 (53.25) | 5629 (46.75) | 6428 (53.25) |
| **Residence** |  |  |  |  |  |  |
| Rural | 9560 (59.99) | 7375 (40.01) | 12082 (57.05) | 9921 (42.95) | 21642 (58.32) | 17296 (41.68) |
| Urban | 3861 (42.65) | 5057 (57.35) | 5500 (44.03) | 6813 (55.97) | 9361 (43.48) | 11870 (56.52) |
| **BMI (kg/m2)** |  |  |  |  |  |  |
| <20 | 5080 (70.67) | 2485 (29.33) | 5669 (67.26) | 3044 (32.74) | 10749 (68.83) | 5529 (31.17) |
| 20-24 | 5871 (55.52) | 5236 (44.48) | 6988 (56.56) | 5632 (43.44) | 12859 (56.08) | 10868 (43.92) |
| 25-29 | 2049 (38.06) | 3168 (61.94) | 3590 (43.58) | 4736 (56.42) | 5639 (41.54) | 7904 (58.46) |
| 30-35 | 291 (30.83) | 636 (69.17) | 1032 (41.18) | 1758 (58.82) | 1323 (38.86) | 2394 (61.14) |
| ≥35 | 48 (33.4) | 109 (66.6) | 240 (23.43) | 544 (76.57) | 288 (24.69) | 653 (75.31) |
| **Smoking Status** |  |  |  |  |  |  |
| No | 5368 (49.8) | 5981 (50.2) | 14238 (52.6) | 13598 (47.4) | 19606 (51.84) | 19579 (48.16) |
| Yes | 8035 (58.44) | 6387 (41.56) | 3334 (54.24) | 3066 (45.76) | 11369 (57.2) | 9453 (42.8) |
| **Alcohol Use** |  |  |  |  |  |  |
| No | 8864 (55.78) | 7943 (44.22) | 16877 (52.84) | 16024 (47.16) | 25741 (53.85) | 23967 (46.15) |
| Yes | 4542 (52.88) | 4433 (47.12) | 697 (54.65) | 647 (45.35) | 5239 (53.05) | 5080 (46.95) |
| **Previously diagnosed DM** |  |  |  |  |  |  |
| No | 12462 (58.85) | 9922 (41.15) | 16595 (56.91) | 13622 (43.09) | 29057 (57.73) | 23544 (42.27) |
| Yes | 957 (26.88) | 2507 (73.12) | 987 (22.52) | 3109 (77.48) | 1944 (24.44) | 5616 (75.56) |
| **Mean (SD) SBP in mmHg** | 117.28 (11.65) | 139.86 (18.05) | 116.51 (11.91) | 137.88 (18.88) | 116.83 (11.80) | 138.70 (18.57) |

Abbreviations: HTN = hypertension; DM = diabetes; SC = scheduled caste; ST = scheduled tribe; OBC = other backward classes; MPCE = monthly per capita expenditure; BMI = body mass index; hypertension; SD = standard deviation; SBP = systolic blood pressure

**Table S3: Predictors of high CVD risk (≥10% risk) (N=58478)**

| **Characteristics** | **Unadjusted OR [95% CI]** | **Adjusted OR [95% CI]** |
| --- | --- | --- |
| **Highest Education** |  |  |
| Not educated/ Up to Primary | Ref | Ref |
| Till Secondary School | 0.93 [0.80, 1.08] | 0.99 [0.88, 1.11] |
| High School | 0.60 [0.46, 0.77] ** | 0.67 [0.55, 0.82] ** |
| College and above | 0.72 [0.60, 0.87] * | 0.72 [0.60, 0.87] * |
| **Marital status** |  |  |
| Never Married | Ref | Ref |
| Currently Married/Cohabiting | 1.49 [0.83, 2.70] | 1.82 [1.05, 3.15] |
| Separated/Widowed/Others | 3.79 [2.08, 6.90] ** | 2.98 [1.70, 5.22] ** |
| **Religion** |  |  |
| Hindu | Ref | - |
| Muslim | 1.09 [0.98, 1.22] |  |
| Others | 1.04 [0.90, 1.21] |  |
| **Ethnicity** |  |  |
| SC | Ref | - |
| ST | 0.93 [0.83, 1.04] |  |
| OBC | 0.96 [0.87, 1.06] |  |
| Other | 1.06 [0.97, 1.16] |  |
| **MPCE Quintile** |  |  |
| Poorest | Ref | - |
| Poorer | 0.95 [0.87, 1.05] |  |
| Middle | 0.99 [0.90, 1.09] |  |
| Richer | 1.03 [0.91, 1.17] |  |
| Richest | 0.90 [0.78, 1.03] |  |
| **Residence** |  |  |
| Rural | Ref | - |
| Urban | 0.92 [0.82, 1.03] |  |
| **Work Status** |  |  |
| Not working | Ref | Ref |
| Currently Working | 0.29 [0.27, 0.31] ** | 0.28 [0.25, 0.31] ** |
| **Alcohol Use** |  |  |
| No | Ref | Ref |
| Yes | 2.18 [2.01, 2.36] ** | 2.20 [1.98, 2.44] ** |
| **Previously diagnosed DM** |  |  |
| No | Ref | Ref |
| Yes | 1.91 [1.62, 2.24] ** | 1.59 [1.37, 1.85] ** |
| **Previously diagnosed HTN** |  |  |
| No | Ref | Ref |
| Yes | 1.89 [1.72, 2.07] ** | 1.63 [1.45, 1.84] ** |

*P<0.05, **P<0.001

P-value for Hosmer-Lemeshow test = 0.23, AUC-ROC = 0.76

Abbreviations: CVD = cardiovascular disease; SC = scheduled caste; ST = scheduled tribe; OBC = other backward classes; MPCE = monthly per capita expenditure; BMI = body mass index; DM = diabetes; HTN = hypertension; OR = odds ratio; CI = confidence interval; Ref = reference

**Supplementary Figure 1: Calibration plot for the multivariable logistic regression model in Table 1**

**Supplementary Figure 2: Calibration plot for the multivariable logistic regression model in Table 2**

**Supplementary Figure 3: Calibration plot for the multivariable logistic regression model in Table 3**
